# A perspective on orchid seed and protocorm development

**DOI:** 10.1186/s40529-017-0188-4

**Published:** 2017-08-04

**Authors:** Edward C. Yeung

**Affiliations:** Department of Biological Sciences, University of Calgary, Calgary, AB T2N 1N4 Canada

**Keywords:** Embryo, Suspensor, Endosperm, Seed coat, Protocorm, Phytohormones, Shoot apical meristem, Regeneration, Protocorm-like bodies, Orchid mycorrhizae, Micropropagation

## Abstract

This perspective draws attention to the functional organization of orchid seed and protocorm during the course of development. The orchid embryos have a well-organized developmental plan generating a blue-print of a protocorm as they mature. The different phases of embryo development in orchids, i.e. histodifferentiation, storage product synthesis and accumulation, and maturation are essentially similar to other flowering plants. The protocorm is considered as a unique structure designed to establish symbiotic association with mycorrhizal fungi and with the primary goal to form a shoot apical meristem. This perspective brings forth arguments that the processes of embryo and protocorm development are highly programmed events, enhancing survival of orchid seeds and plantlets in their natural habitats. Furthermore, the ability of protocorm cells to divide, makes them ideal explants for micropropagation and transformation studies. Through seed germination and micropropagation using protocorms as explants, orchid conservation efforts are greatly enhanced.

## Background

An understanding of plant reproductive biology is essential to plant conservation efforts. In orchids, after a successful pollination event, numerous seeds are produced within a single capsule. Utilizing seeds through asymbiotic and symbiotic seed germination for plant maintenance and propagation are key approaches in orchid conservation efforts. In order to further optimize current methodologies on seed germination and plantlet establishment, we need to have a proper understanding of seed biology, as well as the structure and function of protocorm.

Key features of orchid embryos, seeds, and protocorms are summarized in this article. The globular-shape and small sizes of orchid embryos imply that the embryos are simple and not as well developed as in other flowering plants. On the contrary, the author sees that orchid embryos have developed a plan, generating a blue-print of a protocorm as they mature. The different phases of embryo development in orchids, i.e. histodifferentiation, storage product accumulation, and maturation are essentially similar to other flowering plants. The protocorm formed at the time of seed germination is considered as a unique structure designed to establish symbiotic association with mycorrhizal fungi and with the primary goal to form a shoot apical meristem. This perspective brings forth arguments that the process of embryo and protocorm formation involves highly programmed events, enhancing survival of orchid seeds and plantlets in their natural habitats. The intent of this article is to generate additional interests, discussions, and further studies on orchid seed biology in the near future.

## Embryo development

### The zygote and the proembryo

In flowering plants, a successful fertilization event results in seed formation in which an ovule becomes a seed, the egg cell becomes a zygote, the central cell forms the endosperm, and the integuments develop into the seed coat. Detailed accounts on changes of the egg cell before and after fertilization about orchids are few. In *Epidendrum scutella*, after fertilization, the size of the zygote is similar to the egg cell and there is little change to the vacuolar system within its cytoplasm (Cocucci and Jensen 1969). Rough endoplasmic reticulum and polysomes become more abundant, indicating an increase in metabolic activities within the cell. It is interesting to note that dictyosomes could not be detected in the zygote after fertilization (Cocucci and Jensen 1969). At the light microscopic level, the zygote maintains a uniform dense cytoplasm, e.g. *Cymbidium sinense* (Yeung et al. 1996) and *Calypso bulbosa* (Yeung and Law 1992), or it can have a polarized appearance with a distinct vacuole(s) located at the micropylar end such as in *Cypripedium passerinum* (Law and Yeung 1993) and *Phaius tankervilliae* (Ye et al. 1997). The phenomenon of ‘zygote shrinkage’ (see Natesh and Rau 1984) prior to the first zygote mitosis appears to be absent. Changes in microtubule cytoskeleton have been noted, reflecting dynamic changes within the zygote prior to its first mitotic division. Soon after zygote formation, the microtubules are randomly arranged. As the zygote increases in size, the microtubules organize into a meshwork in the cytoplasm and a dense array of microtubules begins to appear in the cortical region near the chalazal pole (Huang et al. 1998). Moreover, additional studies are needed to decipher cellular events leading to the establishment of cell polarity within the zygote at this critical stage of development.

In the zygote, varying degree of vacuolation appears within its cytoplasm. In *C. bulbosa* (Yeung and Law 1992) and *C. sinense* (Yeung et al. 1996) the cytoplasm of the zygote remains dense through the first mitotic division. In these examples, the first zygote division is usually symmetrical or slightly asymmetrical in appearance. For orchids having a polarized zygote with large vacuole(s) located at the micropylar end of the cell, such as those found in *C. passerinum* (Law and Yeung 1993) and *Phaius tankervilliae* (Ye et al. 1997), the first division is asymmetrical giving rise to two cells of unequal sizes. Changes to the orientation and distribution of the microtubule cytoskeleton are observed at this time (Huang et al. 1998). Irrespective of the cell division symmetry, cell division results in the formation of a terminal and a basal cell, each having a distinct fate. The basal cell can differentiate into a short-lived embryonic organ, known as the suspensor, and the terminal cell will give rise to the embryo proper.

One of the key events occurring after zygote division is the establishment of a polarized embryo axis leading to cell fate determination and the formation of a body plan (Ueda and Laux 2012; Wabnik et al. 2013). Such information similar to the study of *Arabidopsis* is lacking in orchids. The oblique divisions observed in the formation of the proembryo in *C. sinense* (Yeung et al. 1996), the changes in microtubule organization (Huang et al. 1998), the varied patterns of suspensor formation, and limited number of cells in the embryo proper, suggest new regulatory controls in proembryo establishment in orchid.

Based on the cell division pattern in the zygote and proembryo, as well as the varied forms of suspensor morphologies, different embryo types were proposed (Swamy 1949; Johansen 1950). Moreover, the suggested embryological system of classification sees limited use by embryologists. More recently, Clements (1999) provided a more extensive description of embryo developmental types, incorporating the classification system proposed by Dressler (1993).

### The suspensor

The different morphologies of the suspensor within Orchidaceae generate a lot of interest in the study of embryo development (see Yam et al. 2002). The suspensor can appear as a single cell, highly elongated haustorial structures, or multicellular swollen forms that fill the embryo cavity (Swamy 1949). What directs the growth and morphogenesis of the varied suspensor forms? In the study of the nun orchid suspensor development, cytoskeletal elements, i.e. microtubules and microfilaments, play an important role in suspensor morphogenesis (Ye et al. 1997), especially in the elongation of suspensor cells. Dynamic changes in the microtubule cytoskeleton are also observed in *C. sinense* during the course of suspensor formation and elongation (Huang et al. 1998). However, other extrinsic and intrinsic signals regulating suspensor development are not known at present.

With the small size of the embryo proper and the lack of an endosperm, it is logical to speculate that the varied forms of suspensors can play important roles in nutrient uptake. Histochemical staining of cell walls indirectly supports the notion that orchid suspensor can serve as a nutrient uptake site. The polychromatic stain toluidine blue O (TBO) can distinguish lignin, cellulose, and pectic substances on the basis of color differences (O’Brien et al. 1964; Feder and O’Brien 1968; O’Brien and McCully 1981). The fact that the suspensor cell wall stains pinkish-purple with no detectable lignin and cutin deposits, suggests that water and water soluble substances should be able to move apoplastically from the seed coat to the developing embryo proper via suspensor cells (Lee and Yeung 2010). The pinkish-purple color of the suspensor wall after TBO stain also suggests the presence of negatively charged groups which can increase cation-binding capacity of a wall (Smith 1972), and this can aid in solute uptake. A simple plasmolysis experiment indicates that the suspensor cell in the nun orchid has a more negative osmotic potential than the surrounding seed coat cells (Lee and Yeung 2010). The negative osmotic potential provides a driving force for water uptake by the suspensor. In order to visualize the uptake pathway, a fluorescent tracer 6-carboxyfluorescein diacetate (CFD) was applied to developing seeds. Once taken up by the cell, CFD would be hydrolyzed to carboxyfluorescein, giving a fluorescence signal within the cytoplasm. In this experiment, the fluorescence signal was first detected in the suspensor cell before appearing in the embryo proper (Lee and Yeung 2010). This work clearly demonstrates the pathway of solute movement and the importance of suspensor in embryo nutrition.

In flowering plants, the suspensor can take on structural and biochemical specializations (Yeung and Meinke 1993; Kawashima and Goldberg 2010). Transfer cell morphology is one of the common structural specializations observed in suspensor cells. In *Paphiopedilum delenatii*, wall ingrowths are found in suspensor cells (Lee et al. 2006). Wall ingrowths increase the surface area of a cell which can aid in short distance transport. Hence, the formation of wall ingrowths further strengthens the notion that orchid suspensor is specialized in nutrient uptake. The membrane associated with wall ingrowths is unique, e.g. with a higher density of proton pumps (Bouché-Pillon et al. 1994). Future studies demonstrating the presence of biochemical specialization(s) will serve as proof of orchid suspensor function.

In *Phaseolus* species, having large suspensors, high quantities of different phytohormones have been detected (see Yeung and Meinke 1993; Kawashima and Goldberg 2010). Although orchid suspensor is small, it may have a similar biosynthetic ability. If this is true, the orchid suspensor will have additional roles to play during embryogenesis.

Not all orchids have a well-developed suspensor (see Swamy 1949; Clements 1999). In genera such as *Spiranthes*, many species are considered to be suspensorless. At present, the nutrient uptake pathway by these embryos is not known. In the absence of a suspensor, it is logical to speculate that the entire surface of the embryo proper must be able to absorb nutrients directly from surrounding maternal tissues, i.e. the seed coat. In *Cyrtosia javanica*, the seed coat is multilayered and the cells maintain their cytoplasm until seed maturation (Yang and Lee 2014). Furthermore, dense cytoplasmic accessory and antipodal cells are present, locating at the chalazal end of the seed coat, near the developing embryo (Yang and Lee 2014). Can these cells function like the haustoria-type suspensor cells by ‘extracting’ nutrients from the seed coat and further supply them to the developing embryo? In flowering plant, a cuticle is present on the surface of the embryo proper (Rodkiewicz et al. 1994; Lackie and Yeung 1996) and this can impede diffusion of nutrients into the embryo. Moreover, in *C. javanica*, the cuticle has a patchy appearance and it is initially absent from the walls at the chalazal end (Yang and Lee 2014). These structural features indicate that the *C. javanica* embryo is capable of nutrient uptake, especially at the chalazal end near the accessory and antipodal cells. In order to determine how nutrients enter the embryo in the absence of a suspensor, it is necessary to study the microenvironment surrounding the developing embryo and to perform tracer experiments similar to the study for the nun orchid.

### The embryo proper

The pattern of embryo development in flowering plants can be divided into several phases (Goldberg et al. 1994). Once the polar axis is established with the formation of the proembryo, histodifferentiation begins with the formation of a shoot and a root apical meristems, the primary meristems (protoderm, ground meristem, and procambium), and the cotyledon(s). Upon the completion of the histodifferentiation phase, storage product synthesis and accumulation begins within embryo cells. Concomitant with storage product accumulation, the maturation phase sets in and the embryo will acquire desiccation tolerance and prepares for developmental arrest and germination events. This pattern of development contributes to the success and survival of flowering plants in the natural habitats. In lower vascular plants such as bryophytes and ferns, once the histodifferentiation phase is completed, the embryo will germinate immediately without a resting phase, as they do not have the ability to undergo storage product biosynthesis and developmental arrest. Moreover, in flowering plants, given the right conditions, developing embryo can also precociously germinate (Raghavan 1997) after the completion of the histodifferentiation phase, as the embryo has already established a functional body plan ready for germination.

Structural descriptions of mature orchid embryos are detailed by Yam et al. (2002). The number of cells and embryo sizes vary among species of Orchidaceae. Only 8 cells were reported for *Epipogium aphyllum* and approximately 700 cells for *Bletilla striata* (see Yam et al. 2002). The mature *B. striata* embryo appears to be one of the largest in the orchid family. Does histodifferentiation occur within a tiny orchid embryo proper? Although there is no obvious tissue pattern formed, histodifferentiation can still be identified (Vinogradova and Andronova 2002). The following discussion argues that histodifferentiation is an integral and a key step during early embryogeny. In orchid embryos, a gradient of cell size is often recognized with smaller cells located at the apical (chalazal) end and larger cells located at the basal (micropylar) end of the embryo. This represents the existence of a structural polarity. The cells at the apical pole of the embryo are destined to form a meristematic zone and the basal cells are designed to house the symbiont upon seed germination. Since the small and large cells have distinct cell fate upon seed germination, a physiological polarity must also be present within the embryo prior to seed germination. It is interesting to note that for easy-to-germinate species such as *Phalaenopsis amabilis*, a marked gradient of cell size exists within the embryo (Lee et al. 2008). This is in contrast to the difficult-to-germinate species such as *C. bulbosa* in which there is no marked difference in cell size (Yeung and Law 1992). Another indication of histodifferentiation is the formation a protoderm. Judging from the uniform cell profile of the surface layer, the embryo proper has acquired protoderm characteristics. A cuticle is found to envelop the embryo proper such as in *C. sinense* (Yeung et al. 1996) and *P. delenatii* (Lee et al. 2006). The ability to form a cuticle is an indication that the surface cells have acquired epidermal cell characteristics. The best argument that histodifferentiation has indeed occurred in an orchid embryo is that ‘immature’ orchid seeds can germinate asymbiotically in vitro, in a simple medium as long as sucrose is present (Knudson 1922). The orchid embryo has to establish a protocorm body plan during early embryogeny, in order to germinate precociously.

In orchids, the embryo is small with a reduced number of cells. Mitotic activities are arrested early. One can encounter mitotic figures readily within the embryo proper mainly soon after fertilization. Since the embryo proper has few cells, especially when compared to other flowering plants, this suggests that the rate of cell division is slow and/or the cells have a long cell cycle time. A recent study on the expression patterns of the cell-cycle genes in *Phalaenopsis aphrodite* indicates that genes are coordinately regulated from ovule development to embryogenesis (Lin et al. 2014). It would be interesting to determine the intrinsic factors within the embryo which regulate the cell cycle program and mitotic activity as embryo develops.

The absence of a cotyledon is a common characteristic of orchid species. Not more than 10 species have been reported to have a small protrusion that appears to be a rudimentary cotyledon (see Nishimura 1991). In a majority of cases, the protrusions fail to develop further in a protocorm and appear to have no contribution to protocorm development (Nishimura 1991). *Bletilla striata* has the most distinct protrusion at the apical end of the embryo; can this protrusion be considered a cotyledon? It is important to note that *B. striata* has one of the largest embryos in Orchidaceae (Yam et al. 2002). Judging from scanning electron micrographs, the expanding rudimentary cotyledon of *B. striata* closely encircles the developing shoot apical meristem (SAM) (see Figs. 4–12 in Vinogradova and Andronova 2002). Unlike other orchid protocorms, an expanded meristematic zone is not present prior to SAM formation. The close physical proximity between the protrusion and the SAM suggests that it might be a cotyledon. As shown in *Arabidopsis* embryo development, the SAM and cotyledons are formed at the time during histodifferentiation (Laux et al. 2004). If SAM cells or cells having SAM characteristics can be demonstrated in *B. striata* embryo during embryogeny, the protrusion should be regarded as a cotyledon. Future molecular genetics studies on embryo development will provide new insight into the identity of *B. striata* cotyledon-like protrusion and why cotyledons are absent in Orchidaceae.

In flowering plants, storage product deposition begins soon after the completion of the histodifferentiation phase. In dicots such as canola and *Arabidopsi*s, the embryo proper begins to expand and fills the endosperm cavity and mitotic activities soon subside. This is followed by the appearance of starch deposits. Starch granules are subsequently replaced by the formation of storage protein and lipid bodies. In orchids, a similar pattern is observed. In *C. sinense* (Yeung et al. 1996), starch granules first appear within the cytoplasm. The large vacuoles are gradually replaced by small ones and storage protein and lipid bodies begin to appear. In a mature orchid embryo, there are abundant storage protein and lipid deposits; starch granules are rare. Besides histological studies, little is known about the physiology of storage product deposition and how storage products influence germination behavior and long term seed storability (Schwallier et al. 2011; Colville et al. 2016). In canola microspore-derived embryos, abscisic acid (ABA) has been shown to induce lipid and oleosin biosynthesis (Zou et al. 1995). ABA levels begin to increase in *Cypripedium formosanum* (Lee et al. 2015) approximately at the same time of storage product accumulation, indicating that ABA can play a role in storage product deposition in orchids.

Are there sufficient storage reserves to sustain seed germination? Orchid embryos though small, have cells packed with storage products. In their natural habitat, it has been reported that the storage products, especially lipid reserves are not utilized unless mycorrhizal establishment has initiated. Harrison (1977) suggested that “this slow expenditure of reserve metabolites could permit the orchid seedling to survive for a longer period until an appropriate endophytic infection is established”. In *Arabidopsis*, ABA levels can influence availability of energy and nutrients in seeds during germination (Garciarrubio et al. 1997). A high level of ABA can prevent the breakdown of storage proteins (Garciarrubio et al. 1997) and lipids (Graham 2008).

The final stage of embryo development is considered as the maturation phase. Seeds need to be desiccation resistant in order to survive the natural environment. Recent evidence indicates that a desiccation period is necessary, preparing seeds for subsequent germination (Holdsworth et al. 2008; Angelovici et al. 2011). In *P. amabilis* hybrids, the decline in relative water content between 150 and 165 day after pollination coincides with the acquisition of desiccation tolerance (Schwallier et al. 2011). The presence of high levels of ABA in mature orchid seeds may be essential for the acquisition of desiccation tolerance, as demonstrated in the study of alfalfa seeds (Xu and Bewley 1995). In order to respond to potential abiotic stresses, a common biochemical marker, the late embryogenesis abundant (LEA) proteins, are shown to accumulate at the time of seed maturation in *Dendrobium officinale* (Ling et al. 2016). The presence of long lived mRNAs in mature seeds is a well-documented phenomenon in many flowering plants (Dure 1979, 1985; Sano et al. 2012). Recent proteomic analyses in rice indicated the upregulation of 20 proteins even in the presence of a transcription inhibitor, actinomycin D, indicating that long lived mRNAs must be present in mature seeds (Sano et al. 2012). In *Spathoglotis plicata*, Raghavan and Goh (1994) used the ^3^H-poly-(U) in situ hybridization method to demonstrate that poly(A)-RNAs are present and uniformly distributed throughout the mature embryo. This study indicates the potential presence of long-lived mRNAs in mature embryo cells. Future proteomic analysis, similar to that performed in rice, may enable the identification of proteins essential to early stages of germination. From the available information, preparations during seed maturation in orchids appear to be similar to other flowering plants.

The tiny orchid embryo is often described as “globular”, implying that orchid embryo is poorly developed and organized when compare to embryos of other flowering plants. Judging from the cell arrangement and their ability to form protocorms upon germination, histodifferentiation has occurred and the ‘protocorm body plan’ is laid down ready for germination. As seeds mature, additional changes have taken place in preparation for developmental arrest and subsequent germination events. Therefore, one should consider that the pattern of embryo development in orchids is similar to that of other flowering plants; not simple, nor primitive.

### The endosperm

Yam et al. (2002) provided a detailed account summarizing the presence, absence and/or development of an endosperm in orchid embryos. Although some degrees of endosperm development, i.e. with a multinucleate stage in some species (Yam et al. 2002), continual development similar to other flowering plants is absent. The failure to form a functional endosperm is one of the distinctive features in orchid seed development. Why does endosperm formation fail? Anomalous development in the ovules and/or the male gametes can contribute to endosperm failure in orchids. The formation of the central cell in an embryo sac is complex (Lopez-Villalobos et al. 2016). The competition for nutrients among numerous ovules, may have a negative effect on central cell development. Is the reduction in the number of nuclei present within an embryo sac, commonly referred as the ‘striking phenomenon’ (Yeung and Law 1997; Batygina et al. 2003), an early indication of central cell abnormality? In *E. scutella*, the polar nuclei fuse before fertilization; however, fusion is not complete (Cocucci and Jensen 1969). In *C. sinense* a microtubular network can be found in the cortex of the endosperm cell up to the four-cell stage of embryo development; however, microtubules are not found to be associated with the nuclei present (Huang et al. 1998). The lack of a functional cytoskeletal network may be partially responsible for the absence of mitotic activities within the endosperm cell.

Can the male gametes contribute to endosperm failure? In *E. scutella*, once the sperm nucleus enters the central cell, the chromatin appears less dense in the embryo sac than in the pollen tube (Cocucci and Jensen 1969). In the nun orchid, the gamete nucleus that fuses with the polar nuclei of the central cell also has a lower staining intensity, prior to fertilization (Ye et al. 2002). Gamete dimorphism has been reported in sperm cells of a number of species such as tobacco late in sperm development (Tian et al. 2001). Preferential fertilization of one of the sperm cells with the egg has been noted. In *Plumbago zeylanica*, the plastid-rich, mitochondrion-poor sperm cell tends to fuse with the egg cell (Russell 1985) and a preferential transmission of supernumerary B chromosomes to the egg cells during sexual reproduction in maize has been reported (see Weterings and Russell 2004). Although gamete dimorphism is not a universal phenomenon in flowering plants, if there is defect in the male gamete destined for the central cell, endosperm development could fail. In orchids, since a massive number of pollen tubes is present within a capsule prior to fertilization, this is an excellent experimental system to study sperm cell structure and function and the results can provide clues as to whether the male gametes can contribute to endosperm failure in orchids.

A varied number of nuclei is present within the primary endosperm cell after fertilization. The origin of these nuclei has generated a lot of discussion. The nuclei within the endosperm cell can represent unfused nuclei from the central cell together with one of the gamete nuclei, or as mitotic products of the primary endosperm nucleus after fertilization. It is generally agreed that there is no fusion of nuclei within the central cell and the nuclei soon degenerate within the primary endosperm cell as the embryo begins to develop. In *Vanilla planifolia*, Swamy (1947) reported the formation of a primary endosperm nucleus and having several rounds of division prior to degeneration; however, a recent study by Kodahl et al. (2015) indicated that such a process did not occur. It is also possible that “remnants of the primary chalazal nuclei persist in the organized embryo sac” as in *Epipogium roseum* (Arekal and Karanth 1981), contributing to the confusion of nuclear number within the primary endosperm cell. Even with improved optical instrumentation, the origin of various nuclei is difficult to determine. To confirm that the nuclei observed are indeed originated from the primary endosperm nucleus, one needs to clearly demonstrate the presence of mitotic figures within the primary endosperm cell.

The lack of a functional endosperm may be the cause of “tiny” embryo formation. In the majority of flowering plants, after fertilization, endosperm develops rapidly aiding in seed enlargement. The endosperm also serves as a nutrient depot and is also a rich source of phytohormones such as gibberellins and cytokinins. All these factors can promote and regulate embryo development (Lopes and Larkins 1993). In the absence of an endosperm, the orchid embryo cannot expand and hence remains small.

In general, when the endosperm fails to develop, the embryo also aborts (see Vijayaraghavan and Prabhakar 1984). The fact that the orchid embryo continues to develop and survive without the presence of an endosperm indicates modifications to the embryo developmental program. The ability of the suspensor to acquire nutrients and the presence of a cuticle to prevent rapid desiccation, are possible strategies that enable continual embryo development in the absence of an endosperm. The ability of orchid embryos to develop without an endosperm, removes one of the potential barriers for hybrid failure (van der Pilj and Dodson 1966).

One can envisage that orchids have devised a new strategy in seed development during their evolution. Since the success of plantlet formation rests on the success of protocorms in establishing proper mycorrhizal interactions, endosperm formation is deemed unnecessary.

## Seed development-regulation by phytohormones

Phytohormones play important roles in all aspects of plant growth and development. Similar to other flowering plants, phytohormones play important roles in orchids. In *E. ibaguense*, following pollination, changes in phytohormones are noted during fruit and seed development with a continual increase in cytokinin activity and a detectable gibberellin activity, indicating the involvements of phytohormones in orchid fruit and seed development (Taylor et al. 1982). At present, studies on phytohormones focusing on orchid reproductive development are few. More efforts are needed in the future.

### Auxin

In orchids, auxin has been shown to play essential roles during pollination and early stages in fruit development (see Novak et al. 2014). Changes in ethylene and auxin are noted at the time of pollination in *Phalaenopsis*. Ethylene in the presence of auxin is required for initiating ovule development and pollen germination and growth (Zhang and O’Neill 1993). During protocorm development, polar auxin transport inhibitors can influence leaf formation and protocorm morphogenesis (Novak et al. 2014). Expression analysis of fertilization and early embryogenesis-associated genes in *Phalaenopsis* indicate that auxin and ethylene can play a role at the critical stage of seed formation (Chen et al. 2016). Although the precise action of auxin is not clear during seed development and germination, the information at hand indicates that auxin must has an important role to play.

### Cytokinins

Cytokinins are essential to plant growth and development, especially in regulating mitotic activities (Emery and Atkins 2006). In *E. ibaguense*, an increase of cytokinin activity coincides with fertilization and commencement of embryo development (Taylor et al. 1982). Since by that time cell division in the fruit wall has more or less completed, the increase in cytokinin activity is most likely related to embryo development (Taylor et al. 1982). However, unlike the majority of flowering plants, cytokinin activity remains high throughout maturation. Is this a unique phenomenon common to orchids? Do the high levels of endogenous cytokinins play a role in seed germination? Many questions remain to be answered.

### Gibberellins

In seeds such as cereals and legumes, major peaks of biologically active gibberellins are found during early seed development, and little activity can be detected as seeds mature (see Taylor et al. 1982). In *E. ibaguense*, although there is a gradual reduction in GA activities, a significant level can still be detected in mature seeds, similar to the pattern observed for cytokinin activity. Again, this begs the question as to whether gibberellins have a role to play during germination and protocorm development.

### Abscisic acid

It is well documented that abscisic acid (ABA) plays important regulatory roles in different aspects of seed development such as storage product biosynthesis and seed dormancy (Nambara et al. 2010). Endogenous ABA has been reported by Van der Kinderen (1987) using chromatographic methods and by Lee et al. (2007b; 2015) using the technique of ELISA and immunostaining. In *Cypripedium formosanum* (Lee et al. 2015), ABA level remains low during early seed development and rises rapidly mid-way during seed maturation. The ABA level follows changes in water content suggesting changes in water content can serve as an endogenous signal for ABA biosynthesis. The importance of ABA is clearly demonstrated experimentally through the use of the ABA biosynthesis inhibitor, fluoridone. Enhanced germination of mature seeds was noted after injecting fluoridine into developing seed capsules (Lee et al. 2015). For difficult to germinate species, the low percentage of seed germination is attributed to their endogenous ABA content (Van der Kinderen 1987). Lowering of ABA level through seed stratification and pre-treatment procedures prior to seed germination can enhance seed germination (Linden 1992). Hence, in vitro culture of immature seeds, prior to the increase in ABA level, improves successes to asymbiotic germination of orchid seeds.

## The physical environment surrounding the embryo

Orchid seeds are small and dust-like, and can have a variety of surface features, i.e. smooth or sculptured. The seed is air-filled and houses an even smaller embryo. The physical properties of orchid seeds and the biological implications of various characteristics have been detailed in an extensive review by Arditti and Ghani (2000) and a summary of seed structures can be found in the monograph by Dressler (1993). The developmental features of seed coats are discussed by Molvray and Chase (1999) and Lee et al. (2007a).

Although orchid seeds are small, they have distinct seed coat features. In general, the seed coat is composed of a few layers of cells with no vascular tissue present, and it develops mainly from the outer integument of the ovule after fertilization. The inner integument tends to degenerate. Exceptions are noted as in the subfamily Vanilloideae; a number of species, e.g. *Vanilla planifolia* (Swamy 1947) and *Cyrtosia javanica* (Yang and Lee 2014) have a multi- layered seed coat and the outermost layer is heavily lignified. For the majority of orchid seeds, even though the seed coat is thin, the addition of secondary walls, phenolic substances, and cuticular materials offer additional protection to the embryo within.

The inner integument of the ovule usually fails to develop much further after fertilization, the cells often degenerate or collapse around the embryo proper forming a ‘shell’, termed as ‘carapace’ (Veyret 1969; also see Yam et al. 2002). In *Dactyloriza majalis*, a carapace is readily detected and it wraps tightly around the embryo (Rasmussen 1995). The carapace can offer further protection to the embryo. Additional deposition of phenolic compounds and cuticular materials further strengthens the carapace. The low percentage of mature seed germination observed in *Cephalanthera falcata* is attributed to the accumulation of substances such as lignin in the inner integument, a possible cause of germination inhibition (Yamazaki and Miyoshi 2006). In easy-to-germinate species such as *Orchis morio* and *Serapias lingua*, a carapace is incompletely formed around the embryo (Veyret 1969). In the natural environment, the presence of added protection from carapace may prolong seed survival, enhancing their chance of establishing mycorrhizae association with an appropriate symbiont. However, it can be one of the major causes in inhibiting germination of mature seeds in vitro.

## Knowledge of seed development aids in asymbiotic seed germination efforts

The features discussed above enable orchid seeds to germinate symbiotically in their natural habitats. Moreover, features such as high ABA levels and the formation of a thick carapace with phenolic deposits will have negative impacts on asymbiotic seed germination. It is well established that selecting ‘green’ capsules at a certain time of fruit development increases the rate of asymbiotic seed germination. This phenomenon can now be explained by the fact that the embryo has completed the histodifferentiation program with the formation of a blue-print for protocorm formation. Also at this time, ABA level is low and only a thin cuticle is present around the embryo proper. Additional physical barriers such as a thick seed coat and carapace have not yet fully developed. All these features facilitate asymbiotic seed germination of immature seeds.

The accumulation of high levels of ABA and secondary metabolites are recognized as key negative factors in asymbiotic germination of mature seeds. Methods are now available to reduce the levels of inhibitory substances using solvents in conjunction with seed sterilization protocols (Kauth et al. 2008; Lee 2011). Additional methods, i.e. low temperature, including freezing (Rasmussen 1995; Mweetwa et al. 2008), and sonication (Miyoshi and Mii 1988) are some of the methods that can improve asymbiotic mature seed germination (see Lee and Yeung 2017).

Although successes are regularly reported in the literature, it is important to note that when comparing asymbiotic and symbiotic germination of the same species, the development of the protocorms and plantlets can differ (Vinogradova and Andronova 2002) and growth is usually better in the latter (Johnson et al. 2007). This indicates that current in vitro protocols and/or culture media still need to be improved and the conditions optimized.

## Protocorm

Upon seed germination, the orchid embryo first develops into a protocorm before forming a plantlet. The term protocorm was introduced by Treub in 1890 to describe the early stages of in the germination of lycopods (Arditti 1992). Veyret (1974) and Vinogradova and Andronova (2002) gave detailed accounts on the varied forms of protocorms and plantlets in their extensive reviews.

From a developmental and functional point of view, orchid protocorm is a unique structure designed to establish symbiotic association with a compatible fungus, and with the primary goal to form a shoot apical meristem (SAM) for plantlet growth. The body plan of a protocorm is established during embryogeny, with cells having distinct cell fates. Hence the protocorm stage should not be considered as an extension of embryonic development. A recent molecular biology study also indicates that protocorms are molecularly distinct from zygotic embryonic tissues in *Phaleaenopsis aphrodite* (Fang et al. 2016). In order to avoid confusion in description, one should reframe from using the term ‘embryo’ when referring to a protocorm.

Upon placement on appropriate medium, the embryo within the seed coat begins to swell in size and becomes a protocorm. Cells at the apical end divide rapidly, while those at the basal end enlarge and increase their ploidy level during asymbiotic (see Chen et al. 2009) and symbiotic (Rasmussen 1990) germination. The early events in protocorm development are likely regulated by the preprogrammed long-lived mRNAs formed during seed maturation as indirectly demonstrated by Raghavan and Goh (1994). The unique properties of protocorms are further illustrated during symbiotic seed germination. Recent EST analysis in *Dendrodium officinale* reveals the involvement of putative genes (Zhao et al. 2013). Genes and proteins related to defence and stress response, metabolism, transcriptional regulation, transport process and signal transduction pathway are upregulated (Zhao et al. 2013; Valadares et al. 2014; López-Chávez et al. 2016). These studies indicate that the protocorm has the ability to response and prepare to interact with mycorrhizal fungi.

### Protocorm is prepared to receive its symbiont

Fungal hyphae enter the imbibed embryos or developing protocorms at their basal end. For newly imbibed seeds, the degenerated suspensor appears to be the preferred site of hyphae entry (see Wright et al. 2005). Although the suspensor has degenerated, because its wall is primary in nature, penetration through the suspensor or nearby cells is ensured. The presence of the carapace and the cuticular covering of the embryo proper ensure that the apical protocorm cells are protected and not physically damage by the hyphae. For protocorms with more advanced development, i.e. with rhizoid formation, epidermal cells and rhizoids appear to be the preferred site of hyphae entry (see Wright et al. 2005). Stoutamire (1983) suggests that the trichomes may have a function to play in establishing mycorrhizal association.

In *Caladenia tentaculata*, the compatible fungal isolate always infects the seed through the suspensor, resulting in a successful mycorrhizal establishment in protocorms (Wright et al. 2005). In this orchid, phenolic material produced by the embryo diffuses towards its suspensor end; this substance may guide the hyphae towards the embryo. Pelotons appear within the enlarged basal cells of the protocorm and they are digested by protocorm cells at regular intervals for nutrients. For incompatible strains, fungal hyphae do not infect protocorms. They enter through rhizoids or other epidermal cells rather than through the degenerated suspensor. In this case, peloton fails to form and the protocorms are often destroyed by the fungal hyphae (Wright et al. 2005). Moreover, in *Dactyloriza majalis*, fungal hyphae have to enter the protocorm through rhizoids in order to be successful (Rasmussen 1990); entry of hyphae through the suspensor fails to establish mycorrhizal association.

The site of hyphae entry may have important consequences in the success or failure in establishing mycorrhizal association. Since some orchids are ‘suspensorless’ while others may have taken a longer time to germinate forming rhizoids, the subtle structural and physiological differences in protocorm development may determine which symbiont is best suited for a particular orchid. Do different mycorrhizal fungi have their own preferred site of entry into a protocorm in order to establish a successful interaction? When comparing in vitro vs. in situ studies, selectivity toward fungal symbionts differs for the same species (Masuhara and Katsuya 1994; see also Smith and Read 2008). Is that due to minor differences such as the presence or absence of rhizoids in protocorm development?

The ability to digest pelotons to obtain nutrient supplies is an important strategy in orchids in order to achieve successful plantlet formation. For mycotrophic orchids, nutrients have to come from the mycorrhizal fungi. A recent study by Fochi et al. (2017) demonstrated that a bidirectional nutrient transfer can occur between orchids and its symbionts. Future studies using combined techniques of molecular genetics, cell biology, biochemistry, and physiology will provide additional insights and possible mechanisms into nutrient transfer between them. Besides contributing nutrient supplies to the orchid host, mycorrhizal fungi are known to produce phytohormones (Liu et al. 2010). Can they contribute to morphogenesis and subsequent plantlet formation of protocorms through phytohormone supplies? Although there are still many unanswered questions, the above examples highlight the unique properties of protocorms and the process of mycorrhizal establishment and their interactions.

### The ultimate goal of a protocorm is to form a shoot apical meristem (SAM)

**Fig. 1:**
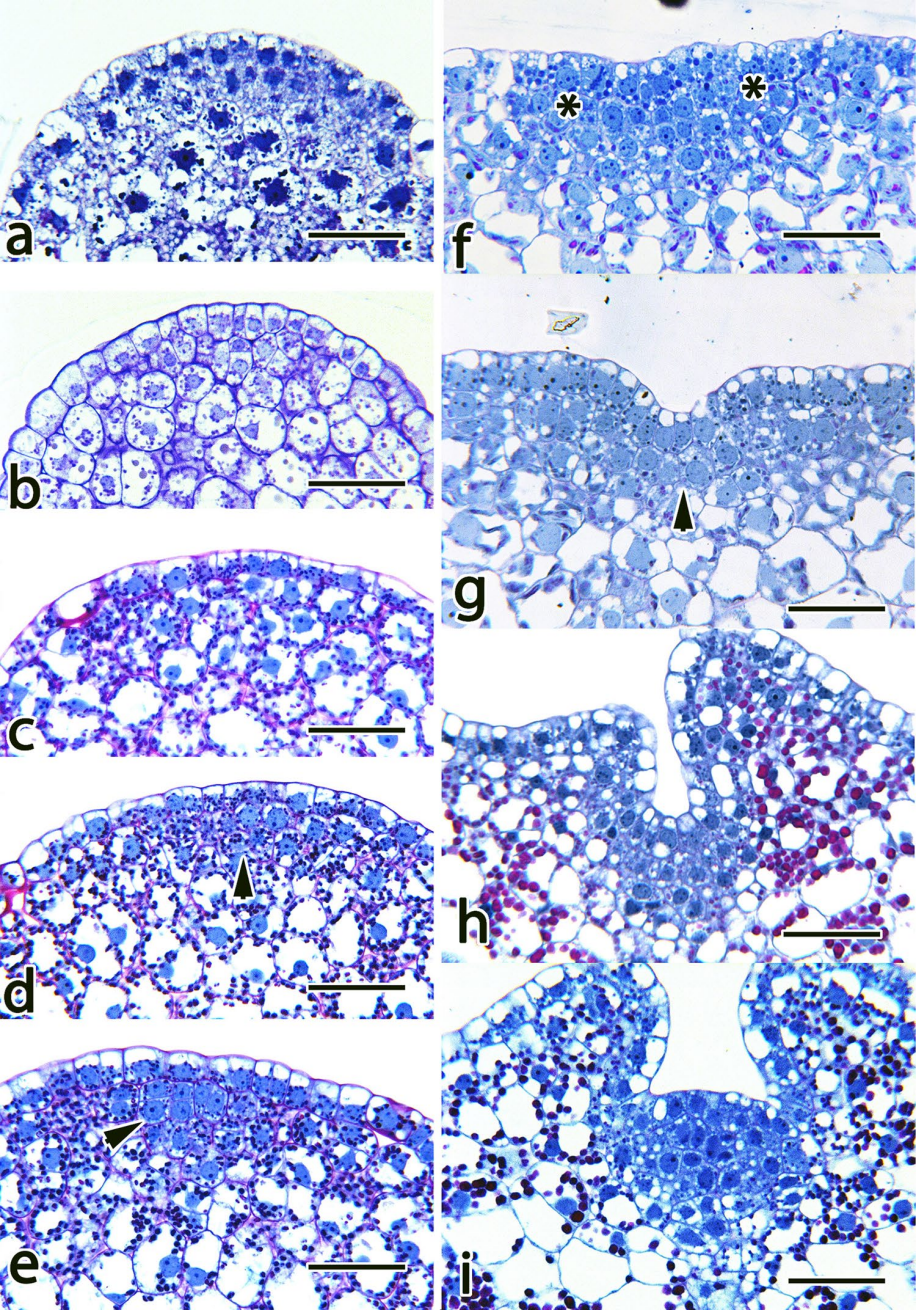
Shows the structural ontogeny of a shoot apical meristem in *Epidendrum ibaguense*. **a** At the time of seed germination, the embryo cells have abundant storage product deposits. **b** Within 96 h, storage products have been mobilized and cells are becoming vacuolated; judging from the cell profile of the apical layer, mitotic divisions have begun. **c** Approximately 7 days after germination, protocorms start to turn green, plastids become abundant with starch within protocorm cells. Protocorm cells at the future shoot pole, albeit smaller, have similar cytological features. **d** Soon after, future shoot meristem cells (*arrowhead*) can be identified as they are smaller in size and with a large nucleus to cytoplasm ratio. **e** The SAM initials (*arrowhead*) take on a *square*-*shape* and starch granules become less abundant. These features clearly mark the structural identity of SAM cells. **f** The SAM increases in size and starch granules remain less abundant when compare to the rest of the protocorm cells. In the peripheral region of the SAM, the cells (*Asterisk*) start to expand. **g** Continual expansion of peripheral meristem cells result in the formation of a protrusion; this results in the formation of a ‘dimple’ with the SAM cells (*arrowhead*) located at the depression. **h** The SAM continues to grow in size, surrounded by a developing leaf primordium. **i** The SAM in a mature plantlet takes on a slightly domed-shaped structure, forming leaf primordia at a regular interval. All *scale bar* = 50 µm

Meristems make the plants (Sussex 1989). Without a functional SAM, there is no further development into a plantlet. Figure 1a–i detail SAM formation in *E. ibaguense*. The embryo at the time of germination has a dense cytoplasm, especially at the future shoot pole. Protein bodies are abundant at this time (Fig. 1a). Within 96 h of germination, storage products are mobilized and judging from the cell profiles, mitotic activities have initiated (Fig. 1b). In *Spathoglottis plicata*, active mitotic activities can be found in the apical pole of the protocorm as early as 4 days after sowing (Raghavan and Goh 1994). With the disappearance of storage products, plastids become abundant within protocorm cells and starch granules are readily detected within plastids (Fig. 1c). The cells at the shoot pole remain small and have similar cytological features (Fig. 1c). Soon after, for cells that are destined to form the future SAM initials, they are characterized by having a large nucleus to cytoplasm ratio (Fig. 1d). The shoot apical meristem initials soon become more distinct as cells begin to take on a square to angular shape and starch granules are not as abundant as surrounding cells (Fig. 1e). These cytological features clearly mark the initiation of a SAM.

The next event is the formation of the first leaf primordium. The apical meristem zone first broadens and in the peripheral region, cells forming the first leaf primordium begin to enlarge (Fig. 1f) and as a result, a leaf primordium begins to protrude above the surface (Fig. 1g–i). This results in generating a small depression with the SAM cells located at the center of the depression (Fig. 1g, h). The ‘dimple’-shaped apex has been reported in the literature, e.g. see figures in Leroux et al. (1997) and Vinogradova and Andronova (2002). The SAM continues to mature and increase in size and becomes a dome-shaped structure, initiating leaf primordia at regular intervals (Fig. 1i). It is only after the formation of a well-defined SAM, adventitious roots begin to form near the SAM endogenously.

The nature and origin of the protrusion from a protocorm is a subject of discussion (Vinogradova and Andronova 2002; Batygina et al. 2003; Yam et al. 2002). Should it be considered as a cotyledon or a leaf? Since a protocorm is a post-embryonic structure, the initial leaf-like protrusion formed at the protocorm stage cannot be regarded as cotyledons (Batygina and Andronova 2000). As shown in Fig. 1, the initiation of the first leaf primordium takes place after the formation of a SAM in its peripheral region, even though the SAM has not yet taken on a mature domed-shaped structure.

Although one can pin-point the location of a SAM in the protocorm, the molecular genetics involve in SAM initiation are not known. In Arabidopsis, SAM genes such as *WUSCHEL* and *CLAVATA*3 are expressed early at the histodifferentiation stage (Laux et al. 2004). The importance of *WUS* in meristem formation and function is well established. In monocot species, such as corn and rice, the orthologues of *WUS* appear to be regulated differently from *Arabidopsis* (Nardmann and Werr 2006). What are the genes involved in SAM formation in orchid protocorm? Do SAM genes regulated differently from the rest of the flowering plants? To truly understand the origin of a SAM and the nature of the first leaf primordium, additional detailed cellular and molecular genetic studies are needed. Many important questions still need to be answered.

### When does the protocorm stage end and plantlet stage begin?

Although the formation of a protocorm leading to plantlet formation is a continuous developmental process, for the ease of description and discussion between studies, the author would like to suggest that protocorm stage ends once a SAM is clearly established and the plantlet stage begins. After SAM formation, the protocorm can degenerate or enlarge forming a ‘corm-like’ structure, taking on varied forms (Veyret 1974; Batygina et al. 2003). The timing of root emergence also differs widely between species. Furthermore, the entire plantlet can remain subterranean for quite some time or elongate rapidly, emerges from the substratum and turns green (see Veyret 1974; Batygina and Andronova 2000; Batygina et al. 2003; Vinogradova and Andronova 2002). With such diverse morphogenetic changes, referring these varied forms as protocorms can be confusing.

For the ease of description and comparative evaluations from different published results, morphological stages of a protocorm or a plantlet in one’s study should be clearly defined. The proposed stages of germination and protocorm development by Zettler and Hofer (1998) are useful schemes detailing changes in seeds during the course of germination and protocorm formation. Vinogradova and Andronova (2002) also recognized five shoot types to describe the complexity of shoots formed by the apical buds of protocorms.

## Protocorms as explants for micropropagation and transformation studies

In a micropropagation system, initiating mitotic activities of an explant and generating cells with ‘organogenic’ or ‘embryogenic’ properties can be a challenge. The ability of apical protocorm cells to divide make them ideal explants for micropropagation and transformation studies. Rapid divisions occur ‘naturally’ at the meristematic zone allowing for better responses to exogenously applied plant growth regulators. Protocorm cells still retain ‘embryogenic’ properties, under appropriate in vitro culture conditions. Cells at the apical end can give rise to protocorm-like bodies (PLBs) directly from the surface of protocorms or indirectly through the formation of organogenic calli. The structural organization and developmental properties of PLBs are similar to protocorms in general. PLBs are considered as ‘somatic embryos’ of orchids. Lee et al. (2013) demonstrated that during PLB formation, there is a transient formation of storage proteins within PLB cells. This clearly indicates that PLBs retain features of zygotic embryo development, albeit transient in nature. In an in vitro environment, PLBs quickly “germinate” and function as protocorms, giving rise to plantlets.

Protocols utilizing protocorms for further micropropagation purposes are readily available in the literature (see reviews by Chugh et al. 2009; Teixeira da Silva 2013). Continual optimization of protocols such as thin section techniques (Teixeira da Silva 2013) and new approaches such as mass culture via bioreactor (Park et al. 2000) will be key to both commercialization and conservation of orchids. Descriptive information concerning PLB initiation from different explants is available in the literature, e.g. Paek et al. (2011); however, mechanistic processes regulating PLB formation are still lacking. Further studies will provide needed information on how the process of PLB regeneration is regulated.

Protocorms and PLBs are excellent explants for transformation studies (Hossain et al. 2013). Plant transformation technologies augment conventional breeding program. Due to the long generation time of orchids, transformation may reduce the time needed to produce a desirable trait. The key event in any transformation protocol is the ability of explants to regenerate after transformation. The ability for protocorms or protocorm sections to generate PLBs, make them ideal candidates for transformation studies. Successes are now reported regularly in the literature using protocorms and PLBs as explants (Phlaetita et al. 2015). One can expect rapid progress in this area of research in the near future.

## Protocorm explants can generate polyploidy plants

Successful polyploid induction can improve plant characteristics. For orchids, polypoid production can improve floral or growth characteristics (Chen et al. 2011; Miguel and Leonhardt 2011). In *Vand*a, embryo cells are diploid maintaining a 2C complement of DNA; however, upon in vitro culture, up to 32C DNA content can be observed in some protocorm cells (Lim and Loh 2003). Chen et al. (2009; 2011) took advantage of the endopolyploidy properties of *Phalaenopsis* protocorm in which basal protocorm cells have a DNA content ranging from 2 to 8C. Through regeneration of PLBs from protocorm thin sections and subsequent selection, polyploid plants can be identified and propagated (Chen et al. 2009; 2011).

Due to the small size and the exposed meristematic surface of protocorms and PLBs, polyploid plants can also be generated using anti-microtubule agents such as colchicine and oryzalin (Miguel and Leonhardt 2011; Chung et al. 2014). After treating with these anti-microtubule agents, followed by selection protocols, desirable polyploid plants can be generated, multiplied and maintained (Miguel and Leonhardt 2011; Chung et al. 2014).

## Conclusions and future perspectives

Although it is minute in size, orchid embryo has carefully planned developmental programs ensuring successful symbiotic seed germination in their natural habitats. The outcome of embryo development is to generate a protocorm which has a unique structural designed for mycorrhizal establishment and a goal to form a shoot apical meristem for plantlet establishment.

At present, successes have been recorded for asymbiotic and symbiotic germination of seeds and micropropagation and transformation studies using protocorms (Chugh et al. 2009; Teixeira da Silva 2013; Lee and Yeung 2017). Many protocols are available which will aid to maintain and propagate important orchid species for further conservation purposes. In recent years, genomic information is becoming available for key commercial species, e.g. Cai et al. (2015), Niu et al. (2016), and Zhang et al. (2016). These studies will provide important data bases to decipher further molecular genetic information. In addition to taking advantage of this information, it is important to combine other approaches such as physiological, biochemical and cell biological methods in studying the seed and protocorm biology in order to gain a comprehensive picture of the entire process.

It is clear from this overview that much more information is needed. Many questions remain. From a developmental biology point of view, orchid embryo and protocorm are excellent and exciting experimental systems for plant biology research. Chances are, we will learn new regulatory mechanisms which will provide new perspectives to plant biology as a whole.
